# Adult Community Perspectives on Improving Rural Youth Access to Mental Health Services: A Participatory Group Concept Mapping Study

**DOI:** 10.1111/jrh.70227

**Published:** 2026-10-09

**Authors:** Janessa M. Graves, Demetrius A. Abshire, Jessica L. Mackelprang, Kailee Parrott

**Affiliations:** ^1^ Department of Family Medicine WWAMI Rural Health Research Center School of Medicine University of Washington Seattle Washington USA; ^2^ Nursing and Systems Science Department College of Nursing Washington State University Spokane Washington USA; ^3^ Biobehavioral Health and Nursing Science Department College of Nursing University of South Carolina Columbia South Carolina USA; ^4^ Department of Psychological Sciences Swinburne University of Technology Melbourne Victoria Australia; ^5^ Department of Chemistry University of Washington Seattle Washington USA

**Keywords:** access, community, equity, mental health, rural

## Abstract

**Background:**

Rural youth face barriers to mental health service access, including provider shortages, stigma, geographic isolation, and financial constraints. Community‐engaged approaches are essential for developing contextually appropriate solutions.

**Methods:**

We used Group Concept Mapping, a participatory research method, to identify strategies for improving mental health service access for rural youth in three counties in northeastern Washington State. Adult community members participated in brainstorming, sorting, and rating potential solutions. Statements were rated on perceived impact and perceived equity dimensions and analyzed to identify thematic clusters.

**Results:**

Analysis yielded eight thematic clusters: Youth Life Skills Development, Community‐Based Training & Education, Community Support Systems, Community Awareness & Engagement, Mental Health Services Delivery & Access, Affordability, School‐Based Mental Health Services, and Capacity‐Building in Schools. The Affordability cluster received the highest ratings for both impact and equity. Notably, 72.7% of statements in the Capacity‐Building in Schools cluster fell in the “go‐zone” (above the overall mean for both perceived impact and perceived equity). Strategies related to de‐stigmatization, mental health education, and workforce development were highly rated across clusters.

**Conclusions:**

Rural stakeholders prioritized school‐based interventions, affordability, and mental health education as key strategies to improve access to youth mental health services. Findings highlight the importance of leveraging existing community infrastructure, particularly schools, and addressing mental health workforce shortages. Ratings reflect adult perceptions; youth were not participants. Future research should include youth perspectives and evaluate the feasibility of implementing these community‐identified strategies.

## 1 Introduction

Mental health is a top health priority for people living in rural areas [1]. While evidence suggests that the prevalence of psychiatric disorders, including mood and anxiety disorders, is higher in urban areas compared to rural areas [2], adverse outcomes, including suicide, are substantially more prevalent in rural areas and have led to calls to action to address rural mental health disparities [2].

Rural communities face numerous barriers to accessing healthcare services, particularly mental health services. As many as 28% of rural residents’ outpatient visits for mental health problems are in primary care settings compared to 14% of visits for urban residents [3], and half as many rural Medicare beneficiaries receive services from behavioral health specialists compared to their urban counterparts [4]. Rural residents’ disproportionate reliance on primary care providers for mental health services is likely due to mental health provider shortages in rural communities. For example, 65% of rural counties in the United States lack a psychiatrist, 47% lack a psychologist, and 81% lack a psychiatric nurse practitioner compared to 27%, 19%, and 42% of urban counties lacking these providers, respectively [5]. Other contributing factors include poor insurance coverage, high poverty rates, and limited access to reliable transportation and fuel [4, 6]. While telehealth improves access to care in some rural communities, the lack of broadband and computer technology compatible with telehealth is yet another obstacle to mental health service access for many rural communities [7, 8].

Barriers to accessing mental health services are compounded for rural youth [9, 10, 11]. Lack of parental support, low treatment motivation, stigma associated with seeking mental health care, preference for informal support, limited knowledge about where or how to access services, and lack of local providers who are skilled in working with children and adolescents are among the challenges that rural young people commonly face [11, 12]. Innovative efforts to improve mental health service access and utilization are imperative for this population [13, 14, 15, 16]. Prior to the COVID‐19 pandemic, suicide rates among rural youth were higher than urban youth, and this disparity was widening over time [16]. The pandemic exacerbated disparities in access to care and mental health outcomes [17]. While telehealth served as a stopgap measure during the pandemic, proportionately fewer rural youth have access to technological resources that facilitate telehealth service delivery compared to their urban peers [8].

Given the variety of barriers that prevent rural youth from accessing and using mental health services, it is crucial to collaborate closely with rural communities to identify possible future interventions [18, 19]. There is no one‐size‐fits‐all solution, and paths toward addressing mental health access challenges should be tailored to the context, values, resources, and community culture [20]. Brainstorming solutions and developing interventions with rural communities is therefore necessary for addressing the mental health needs of rural youth. Participatory approaches shift decision‐making toward communities that have often been excluded from research processes and can generate strategies that better align with local resources, institutions, and culture, potentially supporting their sustainability beyond a research program [18, 19, 20].

The purpose of this study aimed to engage and empower members of rural communities to identify approaches to improve access to mental health services for young people in those communities. This work was part of a funded research program and follows our prior studies on the barriers rural youth face in these same counties [9, 10]. Having documented the challenges youth and their families face in accessing care, we turned to the solutions community members themselves proposed to address these challenges.

## 2 Methods

This study used Group Concept Mapping (GCM), a participatory research approach that combines qualitative and quantitative methods, to identify ways to improve access to mental health services for rural youth. GCM is appropriate for use in rural and community settings and prioritizes the inclusion of diverse perspectives and ideas [21, 22, 23].

### 2.1 Study Participants

Adult participants (>17 years) were recruited from rural communities across three counties in northeastern Washington State. Potential participants were identified using snowball sampling and via contacts in the local community, health organizations, and schools. We sought to recruit adult participants who work with or care for youth, such as school personnel or healthcare providers. Participants also included parents. Inclusion criteria included interest in participating in a study about access to mental health services for rural youth and working or residing in one of three target counties. The recruitment counties, classified as rural by the Washington State Department of Health [24], comprise 67,000 residents across 6000 square miles [25]. All three counties are classified as mental health professional shortage areas by the Health Resources and Services Administration [26]. These counties were selected because each is rural and a mental health professional shortage area, and because our prior work there had established relationships with schools, local health jurisdictions, and community organizations [9, 10], and because the funded scope of the project was limited to this region.

### 2.2 Community Advisory Group

A community advisory group was convened for the project, comprised of representatives of local health jurisdictions, school districts, healthcare organizations, parents, and community‐based organizations across the three counties where the study was conducted. Community advisory group members (also referred to as “community partners”) were identified through established connections in the area and especially engaged study participants who expressed interest in partnering on this project.

### 2.3 Study Procedures

GCM involves several key stages that require participant engagement: brainstorming, sorting, and rating.

#### 2.3.1 Brainstorming

A list of potential factors that may improve mental health access for rural youth was generated through a 5‐min online survey (Qualtrics). Data were collected between October 5 and November 15, 2021. Participants were asked:

1. “What are the main emotional problems or concerns that affect children and adolescents in your community?”
2. “List all of the things you can think of that can help children or teens in getting care or support for these concerns in your community.”

We asked participants to be specific in their responses and to avoid general, broad statements. Potential solutions for improving mental health access for rural youth were derived from responses to the second question. Two study team members (JG, KP reviewed all brainstormed items to synthesize responses, which included removing redundant responses, combining similar statements, separating complex statements with more than one idea, simplifying verbose responses, removing inapplicable responses (i.e., “n/a” or “none”), and editing for grammatical and spelling errors. The community advisory group supported recruitment and reviewed the synthesized list to confirm that it reflected the responses received. The final synthesized list of statements was used in subsequent sorting and rating stages.

#### 2.3.2 Sorting

Participants in the sorting and rating stages were recruited from a subset of the brainstorming survey respondents who indicated they were interested in contributing to other aspects of the study and through additional snowball sampling within the counties of interest. Snowball referrals came from brainstorming respondents and from community partners supporting the project. The percentage of participants identified through each mode of recruitment was not recorded. These stages took place from August 13 to September 2, 2022, and were conducted using GroupWisdom (Concept Systems), an online software designed to collect and analyze data from GCM studies.

Sorting involves organizing statements into conceptually similar groups. Participants could create as few or as many groups as desired, so long as they were grouped based on similarity (as perceived by participants) and no group consisted of only one statement. We asked participants to provide a descriptive title or name for each group they created. To guide their sorting, participants were provided the focus prompt, “Children and adolescents living in rural communities face many challenges in accessing mental health services locally. Things that can improve youth access to mental health services include…”

#### 2.3.3 Rating

Following the sorting task, we asked participants to rate each statement in terms of how much impact it would have on increasing youth access to mental health services in their community. Participants were also asked to rate each statement in terms of how equitable the strategy would be in increasing youth access to mental health services. Both rating scales ranged from 1 (*little/no impact; not at all equitable*) to 5 (*very large impact; very equitable*). Prompts for rating questions are provided in Table S1. Impact and equity were selected as rating measures in consultation with the community advisory group as measures of the benefit participants expected a strategy to produce (impact) and whether they expected that benefit to reach youth across the community (equity).

### 2.4 Analysis

We analyzed sorted and rated data using GroupWisdom. Multidimensional scaling was used to generate a spatial point map array of statements across all participants [27]. We assessed the goodness‐of‐fit of the point map by measuring how well the spatial map corresponded to the underlying similarity matrix using an f‐stress value. Acceptable f‐stress values fall between 0.10 and 0.35, with lower scores indicating stronger alignment [23, 28]. Participants’ sorting data were excluded from the analysis if they sorted fewer than 75% of the statements [29].

Next, we used hierarchical cluster analysis to generate a concept map, which involves agglomerative clustering of proximal statements to successively larger clusters based on the sorting data produced by participants [30]. The cluster map illustrates how similar statements are grouped together into clusters. We determined the final number of clusters together with the community advisory group. Together, we compared potential cluster solutions, which ranged from 4 to 10 clusters. We examined how statements split and merged across adjacent solutions, whether every cluster could be labeled in a way partners recognized as describing a real feature of or phenomenon in their communities, and cluster bridging values as an indicator of within‐cluster homogeneity. Statistical indicators helped narrow the candidate cluster set, then we relied upon community partners’ judgment about interpretability and local relevance. Disagreements about a label or the placement of a statement were discussed until agreement was reached. Cluster bridging values range from 0 to 1, with lower values indicating greater cohesion among statements within a cluster and higher values indicating greater heterogeneity. Statement bridging values, also ranging from 0 to 1, indicate how well‐anchored a statement is in its specific location on the point map [23]. Lower statement bridging values reflect statements that were more frequently sorted with other statements located proximally on the map (compared to higher statement bridging values). Higher statement bridging values suggest less consistency in sorting with nearby statements.

Mean rating scores (*μ*) were calculated for statements and clusters (ranging from 1 to 5) for both measures of impact and equity. These data were compared using a bivariate scatterplot called a “go‐zone map,” which illustrates the relationship between both rating scores. The “go‐zone” is defined as the quadrant where statements have mean scores greater than the overall mean for both impact and equity, indicating ideas that participants rated as both highly impactful and highly equitable. A comparison of mean rating scores within each cluster was depicted using ladder diagrams.

Following cluster identification, we held two online sessions with community members, including parents/guardians, public health officials, and local policymakers. Attendees were drawn from the community advisory group. Seven group members subsequently reviewed the final concept map and findings in a virtual meeting. These sessions enabled participants to ask questions, challenge interpretations, and provide critical insights into how the results aligned (or not) with their lived experiences in rural communities. Discussions were documented through written notes rather than video or audio recordings, which we reviewed and integrated into our final interpretation of the concept maps and rating data. Points where community members’ interpretations diverged from one another or from the research team were recorded in these notes and carried into the interpretation below rather than resolved in favor of one reading.

Descriptive statistics were used to describe participant demographics. All participants provided informed consent prior to engaging in the brainstorming or sorting/rating stages. The Washington State University Institutional Review Board approved the study procedures.

## 3 Results

A total of 172 participants completed the survey, yielding the brainstormed items needed for the first GCM stage. Most participants identified as parents or guardians (63.4%) and women (75.0%; Table 1). Participants were evenly distributed across age groups, with most aged 31–40 years (29.7%) and 41–50 years (22.7%). An initial brainstormed list of 475 statements was synthesized into a final list of 79 statements. Table S2 provides the final, synthesized, unsorted list of statements generated during the brainstorming stage.

**TABLE 1:** Characteristics of participants in brainstorming, sorting, and rating data collection stages of study.

	Brainstorming (*N* = 172)	Sorting (*N *= 21)	Rating (*N* = 3)
Age (years)			
18 to 30	38 (22.1)	8 (38.1)	16 (48.5)
31 to 40	51 (29.7)	3 (14.3)	3 (9.1)
41 to 50	39 (22.7)	7 (33.3)	10 (30.3)
51 to 60	25 (14.5)	2 (9.5)	3 (9.1)
Over 60 years	19 (11.0)	1 (4.8)	1 (3.0)
Years living in the rural community			
5 or fewer years	^a^	1 (4.8)	2 (6.1)
6 to 10	^a^	2 (9.5)	5 (15.2)
11 to 20	^a^	10 (47.6)	16 (48.5)
21 to 30	^a^	4 (19.0)	5 (15.2)
Over 30 years	^a^	4 (19.0)	5 (15.2)
Parent or guardian of child(ren) under age 18 years			
Yes	109 (63.4)	^a^	^a^
No	63 (36.6)	^a^	^a^
Role in community (not mutually exclusive)			
Community member	115 (56.1)	^a^	^a^
School personnel	42 (20.5)	^a^	^a^
Healthcare provider	18 (8.8)	^a^	^a^
Other^b^	30 (14.6)	^a^	^a^
Gender			
Woman	129 (75.0)	^a^	^a^
Man	41 (23.8)	^a^	^a^
Non‐binary	1 (0.6)	^a^	^a^
Other^c^	1 (0.6)	^a^	^a^

^a^ Question not asked.

^b^ Other roles included those working in the medical or education fields, social work and social services, and library or education.

^c^ One individual identified as genderfluid.

In total, 21 and 33 participants completed the sorting and rating stages, respectively. Most participants who completed the sorting and rating stages had lived in one of the three participating counties for over 10 years. Rating‐stage participants were younger than brainstorming participants (48.5% vs. 22.1% were aged 18 to 30 years, respectively); parental status, community role, and gender were not collected at these stages (Table 1). Participants sorted statements into 3 to 12 conceptually similar groups (mean [*μ*]: 7.1, median 7). After analysis, a point map was generated, with a stress value of 0.2644, within the acceptable range for interpretable results (Figure S1). There was substantial overlap across the clusters, making it challenging to choose an optimal cluster solution. After comparing solutions ranging from 4 to 10 clusters, we collaborated with the community advisory group to select an 8‐cluster solution based on statistical indicators, cluster interpretability, and local relevance (Figure 1). Cluster bridging values ranged from 0.07 (Cluster 8) to 0.51 (Cluster 4), with a median of 0.28. Brief descriptions of each cluster, along with their respective statements, are reported in Table 2.

**FIGURE 1:**
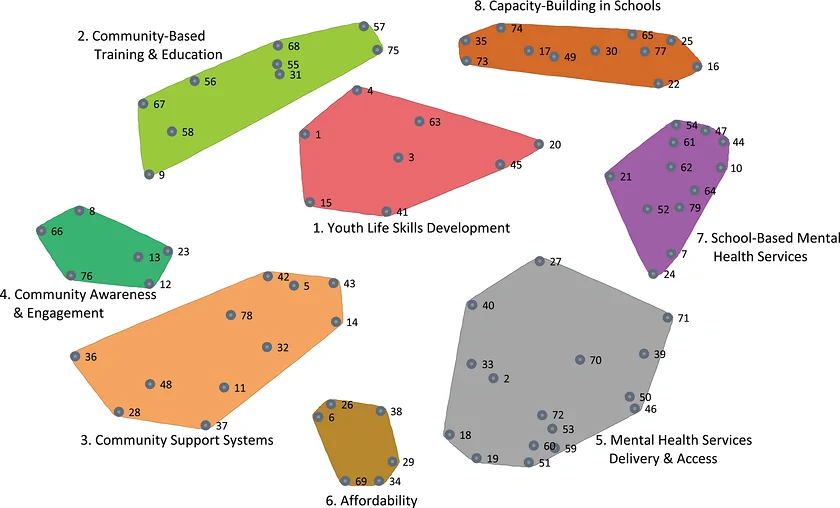
Cluster solution with conceptual groupings of participant‐sorted statements describing potential solutions to improve youth access to mental health services in rural northeastern Washington State. Gray numbered points on this map illustrate two‐dimensional spatial relationships among the 79 statements.

**TABLE 2:** Rating and connections between statements and clusters describing potential solutions to improve mental health services access for youth in rural northeastern Washington State, 2022.

Cluster and statements		Bridging value^a^	Impact rating^b^	Equity rating^c^
**Cluster 1—Youth Life Skills Development**: Focuses on personal development, life skills, and support systems for youth outside formal education settings, including at‐risk and special populations.		**0.23**	**3.93**	**3.72**
1	Assist youth in setting attainable life and emotional goals	0.25	4.00	3.85
3^d^	Continue to educate and support students after graduation	0.18	4.15	3.94
4^d^	Destigmatize mental health by creating spaces for kids and adults to talk about it	0.30	4.27	4.06^e^
15	Ensure extra support for LGBTQ+ students	0.33	3.79	3.55
20	Improve and expand anti‐bullying programs	0.17	3.88	3.63
41	Offer a public speaker forum to touch on mental health in schools and community	0.23	3.00	3.18
45	Offer extra attention and management of at‐risk children	0.18	4.00	3.55
63^d^	Provide life skill training	0.22	4.33^e^	4.00
**Cluster 2—Community‐Based Training & Education**: Centers educational initiatives for families and communities, focusing on relationships, mental health awareness, and preventive education.		**0.33**	**4.09**	**4.00**
9^d^	Educate the entire community about mental health (not just families with kids)	0.38	4.18	4.42^e^
31^d^	Increase awareness on domestic violence, pregnancy, and sexual assault in sexual education	0.23	4.27	4.00
55	Provide education about addiction and how to combat it	0.25	3.97	3.81
56	Provide education about how to foster and protect healthy relationships	0.35	4.00	3.94
57	Provide education on improving social‐emotional skills	0.16	3.91	4.16
58	Provide families with management help and education	0.50	3.85	3.94
67	Provide parents skills to help them be more supportive of and for their kids	0.58	3.97	3.97
68	Provide teen mental health training (e.g., Mental Health First Aid)	0.32	4.30	3.81
75^d^	Teach youth what unhealthy relationships look like	0.15	4.33^e^	3.97
**Cluster 3—Community Support Systems**: Encompasses community‐based support systems including faith‐based, multi‐generational, and alternative programs for youth.		**0.40**	**3.90**	**3.74**
5	Develop and offer more youth‐focused programs in communities	0.14	4.15	3.91
11	Employ more youth advocates	0.41	3.79	3.88
14	Enhance multi‐generational support in the communities	0.13	4.03	3.67
28	Increase anonymity when seeking services	0.66	3.97	4.24^e^
32	Increase church and faith‐based support networks	0.20	3.12	2.82
36	Increase pay for counselors and mental health staff	1.00	3.88	3.73
37	Increase support from government agencies (CPS, DSHS)	0.41	3.70	3.61
42	Offer age‐appropriate support groups	0.23	3.79	3.70
43	Offer alternatives for juveniles involved in criminal justice system (e.g., join a sport, join a mentor program, community service)	0.19	3.97	3.79
48	Offer mental health assistance to youth via text	0.73	4.06	3.64
78^d^	Treat and recognize mental health/illness the same as physical ailments	0.29	4.39^e^	4.21
**Cluster 4—Community Awareness & Engagement**: Emphasizes positive representation, community engagement, and public awareness of mental health resources.		**0.51**	**3.97**	**3.94**
8	Disseminate positive examples and representation of/for those getting help	0.52	3.85	4.03
12	Encourage healthy activities within communities	0.35	3.91	3.91
13	Encourage more physical exercise in the community	0.35	3.79	3.79
23^d^	Improve community awareness of what help is available	0.29	4.30^e^	4.15^e^
66	Provide more information about mental health via social media platforms	0.85	3.88	3.70
76^d^	Train law enforcement in mental health	0.72	4.12	4.06
**Cluster 5—Mental Health Services Delivery & Access**: Addresses physical, financial, and logistical barriers to accessing health services, including transportation and infrastructure.		**0.25**	**4.14**	**4.00**
2^d^	Build community health centers where anyone can receive any form of health care in one place	0.16	4.55	4.13
18^d^	Improve access to basic services for families (housing, clothing, food)	0.18	4.24	4.06
19	Improve access to broadband internet for telehealth	0.23	3.82	4.00
27^d^	Increase access to training on mental health signs and symptoms	0.30	4.21	3.94
33^d^	Increase community resources for mental health	0.18	4.55	4.42^e^
39	Integrate mental health checks in annual child checkup/physical	0.37	4.15	3.64
40	Introduce a mentorship program for youth	0.21	3.79	3.61
46^d^	Offer local, 24/7 counseling services	0.32	4.36	4.41
50	Provide a crisis hotline in local Emergency Rooms	0.39	3.70	3.70
51^d^	Provide access to services regardless of insurance type	0.22	4.58^e^	4.39
53^d^	Provide better/free transportation	0.20	4.12	4.06
59	Provide free bussing to health clinics	0.22	3.91	3.94
60^d^	Provide free health services, including mental health	0.18	4.48	4.42^e^
70^d^	Staff mental health clinics with providers who are trained to work with youth	0.25	4.39	4.09
71	Staff providers at locations where kids frequent	0.43	3.67	3.42
72	Take away the uncertainty (more/better scheduling)	0.18	3.70	3.72
**Cluster 6—Affordability**: Targets financial barriers through funding, staffing, and cost reduction strategies.		**0.31**	**4.27**	**4.17**
6^d^	Develop hotlines for diverse mental health needs (not just suicide)	0.40	4.15	4.36
26^d^	Improve trust between patients and providers	0.24	4.12	4.06
29^d^	Increase availability of help	0.30	4.64^e^	4.39^e^
34	Increase funding for services and to existing organizations	0.32	3.97	3.91
38^d^	Increase the healthcare workers in the community	0.23	4.33	4.03
69^d^	Reduce the costs of services (increase affordability)	0.40	4.39	4.27
**Cluster 7—School‐Based Mental Health Services**: Direct mental health services and programs delivered in school settings.		**0.19**	**3.94**	**3.78**
7	Develop programs for peer‐peer support or peer listening	0.40	3.58	3.64
10^d^	Employ more (or any) mental health counselors at schools	0.21	4.55^e^	4.33^e^
21	Improve communication and cooperation between schools and communities	0.25	3.91	3.94
24	Improve mental health resources for homeschool students	0.29	3.91	3.82
44	Offer external programs, trips, or experiences through schools	0.16	3.79	3.61
47	Offer mandatory one‐on‐one counseling in school (e.g., twice a year)	0.11	3.56	3.44
52	Provide after‐school programs for youth	0.20	4.21	3.91
54	Provide counseling services for teachers and school staff	0.15	4.03	3.88
61	Provide grief counseling through schools	0.07	4.09	3.76
62	Provide group therapy in schools	0.09	3.48	3.33
64	Provide mental health support to youth during school breaks	0.21	4.12	3.72
79^d^	Develop and support school‐based health clinics	0.18	4.09	4.00
**Cluster 8—Capacity‐Building in Schools**: Addresses training, awareness, and educational content needs to enable school staff to support student mental health.		**0.07**	**4.18**	**3.99**
16	Formally train school staff in mental health screening	0.14	4.27	3.82
17^d^	Help students more easily identify their need(s) for help	0.06	4.30	3.97
22	Improve communication and relationships between the school and families	0.16	3.84	3.73
25^d^	Improve school staff ability to recognize and help students with their mental health problems	0.04	4.15	4.12
30^d^	Increase awareness about available support in school	0.02	4.18	4.18^e^
35^d^	Increase mental health education for young children	0.10	4.06	4.00
49^d^	Offer mental health courses in school	0.03	4.27	3.97
65^d^	Provide more information about mental health through schools	0.00	4.33^e^	4.03
73^d^	Teach children coping skills	0.07	4.25	4.12
74^d^	Teach stress management skills to youth	0.09	4.33^e^	4.15
77	Train school staff in trauma informed care or “Trauma Lens”	0.07	3.97	3.82

Abbreviations: CPS, Child Protective Services; DSHS, Department of Social and Health Services (state agency); LGBTQ+, lesbian, gay, bisexual, transgender, queer, and other sexual and gender minority.

^a^ Bridging value: Bridging values are listed for each cluster and statement.

^b^ Impact rating: Average participant rating in response to the following question, “How much impact would this strategy have on increasing youth access to mental health services in your community?” (scale from 1 [*little or no impact*] to 5 [*very large impact*]); listed for each cluster and statement.

^c^ Equity rating: Average participant rating in response to the following question, “How equitable would this strategy be on increasing youth access to mental health services in your community?” (scale from 1 [*not at all equitable*] to 5 [*very equitable*]); listed for each cluster and statement.

^d^ Go‐zone statement (mean rating above the overall mean for both perceived impact and perceived equity) across all participants.

^e^ Statement has highest rating in impact or equity within cluster (most impactful or most equitable solution within cluster).

Overall, participants rated statements with a mean score ranging from 3.00 (“Offer a public speaker forum to touch on mental health in schools and community”) to 4.64 (“Increase availability of help”) for impact (mean impact score overall: 4.05). The statement with the lowest mean score for equity was “Increase church and faith‐based support networks” (*μ* = 2.82). Three statements tied for the highest mean equity rating (*μ* = 4.42). These statements focused on increasing community resources (33), providing services for free (60), and educating the community about mental health (9). The mean equity score across all statements was 3.91. Ratings were compressed toward the upper end of both scales. Statement means ranged from 3.00 to 4.64 for perceived impact and 2.82 to 4.42 for perceived equity; only one statement fell below the scale midpoint on either dimension.

Cluster 6 “Affordability,” which focused on solutions that address financial barriers that hinder access to care, was rated highest both in terms of impact (*μ* = 4.27) and equity (*μ* = 4.17). Most statements in Cluster 6 (83.3%) were in the go‐zone (Figure 2), with mean impact and equity rating scores above the mean. Overall, 72.7% of statements in Cluster 8 “Capacity‐Building in Schools,” which provides suggestions for improving youth mental health access through equipping school staff to support student mental health, were in the “go‐zone.” In contrast, only one statement (9.1%) was in the “go‐zone” from Cluster 3 “Community Support Systems,” which considered community‐based approaches to building supports for youth (Table 2).

**FIGURE 2:**
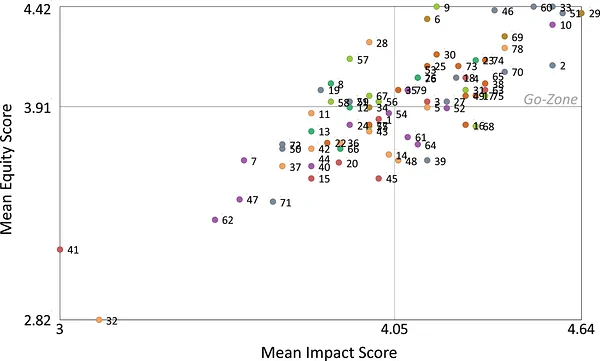
Go‐zone map showing statements rated according to impact (*X*‐axis) and equity (*Y*‐axis) for potential approaches to improving youth access to mental health services in rural northeastern Washington State.

## 4 Discussion

This community‐engaged study sought to identify potential approaches to improve access to mental health services for rural youth in northeastern Washington State. Using a participatory research method, GCM, eight thematic clusters were generated from community input: Youth Life Skills Development, Community‐Based Training & Education, Community Support Systems, Community Awareness & Engagement, Mental Health Services Delivery & Access, Affordability, School‐Based Mental Health Services, and Capacity‐Building in Schools.

The prominence of school‐based solutions was a particularly noteworthy finding. Two distinct but complementary clusters were identified: School‐Based Mental Health Services (Cluster 7) and Capacity‐Building in Schools (Cluster 8), with 16.6% and 72.7% of statements, respectively, falling in the go‐zone (i.e., rated high in both impact and equity). This finding suggests that community members recognize schools not only as sites for direct service delivery but also as critical venues for prevention, early identification, and ongoing mental health support [31]. The emphasis on capacity‐building, including training school staff to recognize mental health problems, increasing awareness of available supports, and teaching coping skills, reflects recognition that sustainable change involves relationship‐building and development of infrastructure rather than solely adding services [32].

Community Support Systems (Cluster 3) received lower ratings, with only one statement (9.1%) in the go‐zone. “Increase church and faith‐based support networks” received the lowest equity rating overall (*μ* = 2.82). Prior research has suggested that rural church congregations are less likely to offer mental and behavioral health programs than urban or suburban congregations [33]. At the same time, rural residents may favor religious institutions and faith leaders over traditional mental health providers for support with emotional distress [33, 34]. Evidence from a recent systematic review suggests that community‐based mental health programming in rural settings, including programming delivered through faith communities, can be feasible [35]. The discrepancy between low‐equity ratings and evidence supporting faith‐based approaches in underserved rural communities may have several explanations. Participants may have judged faith‐based networks as inequitable if support delivered through a congregation is perceived to primarily reach youth affiliated with that congregation or who share its faith, with those outside the church or faith affiliation having limited access to those supports. Alternatively, the rating may reflect sociocultural differences across rural US regions or our recruitment through schools and health organizations rather than congregations. Our data cannot adjudicate between these explanations, and additional research is warranted to further understand the role of faith‐based organizations in addressing mental health needs in rural communities. Nevertheless, the perception of inequity suggests that faith‐based programming may need to be paired with other points of access, including secular or cross‐faith settings.

Multiple statements focused on de‐stigmatization and mental health education received high ratings across clusters. Highly rated statements included educating the community about mental health, creating spaces for children and adults to talk about mental health, and treating mental health with the same priority as physical health. The frequency of these statements across high‐rating categories suggests that stigma may be a salient concern among participants, a finding consistent with previous research in the same geographic area [9, 10]. Generally speaking, stigma represents a widely recognized barrier to mental health service receipt for youth, and there are unique implications in rural communities where privacy concerns and lack of anonymity in close‐knit communities exacerbates obstacles to seeking care [9, 36, 37]. Evidence‐based interventions to reduce stigma, including social contact approaches and co‐designed community education with persons with lived experience of mental health conditions, have demonstrated effectiveness in decreasing stigma [38].

Specific statements that participants rated highly for both impact and equity, such as employing more mental health counselors in schools, providing access to services regardless of insurance type, and teaching stress management skills, represent promising starting points for developing future interventions to better support rural youth. These priorities align with findings from adolescents in rural communities, who identified mental health literacy and mental healthcare workforce issues as primary barriers to care access [37]. Several strategies may enhance implementation feasibility in these settings: using existing infrastructure, particularly schools; training school staff and law enforcement in mental health screening, trauma‐informed care, and crisis response; and expanding telehealth alongside broadband investment. Participants emphasized the need for providers trained to work with children and adolescents, a known disparity in rural communities [5], underscoring the importance of tailored workforce development efforts to better serve rural youth mental health needs.

It is important to note that go‐zone statements varied in their readiness for implementation. Some named a specific service in a defined setting and correspond to strategies with an established evidence base, such as employing mental health counselors in schools and developing school‐based health clinics [32] or community education paired with contact‐based approaches to reducing stigma [38]. Other statements were directional, identifying an outcome community members want without specifying who would act, in what setting, or by what mechanism. For example, several statements in the Affordability cluster describe desired outcomes rather than specific financial strategies for improving access to mental health services. This variation is a feature of the GCM process rather than a shortcoming of the responses. Participants were asked to identify “things that can improve youth access to mental health services,” which could range from broad priorities to specific programs. A logical next step is to work with communities to identify evidence‐based interventions that align with the priorities identified through GCM and to assess their appropriateness, acceptability, and feasibility within the local context. Local health jurisdictions, school districts, and community advisory groups can help lead this process. Our team recently applied a similar approach with The Rural Network, a statewide coalition of parents, school staff, and local leaders, to identify potential interventions aligned with GCM‐identified risk and protective factors for youth substance use in rural communities [39].

## 5 Limitations

This study has several limitations. First, the project's scope was geographically limited to three counties in northeastern Washington State. While this circumscribed focus was a logistical necessity due to funding constraints, some participants identified potential facilitators to improving youth mental health access that may be unique to their particular community. Many of the approaches identified are nonetheless relevant to rural communities elsewhere in the United States, particularly those related to affordability, school‐based services, and workforce development, though the relative impact or equitability of specific solutions may vary across rural contexts.

Second, the approaches brainstormed, sorted, and rated in this study were identified exclusively by adults and do not include youth voices. While adults may be better positioned to implement interventions and may observe barriers from multiple perspectives [9, 10], youth themselves possess critically important insights into their experiences, needs, and preferences that were not captured in this study. Adult‐identified priorities may diverge from youth preferences in specific ways. The prominence of school‐based solutions may reflect adults’ familiarity with schools as an institution through which they can reach youth, as much as youth's preference for seeking help there. Yet, youth frequently report concerns about confidentiality and about being seen seeking help, concerns that can be amplified in a small school in a small community [11, 12, 37]. Consistent with this, mental health assistance via text and peer support programs, two statements in this study that may be arguably most similar to the types of confidential and peer‐based supports adolescents often prefer [40, 41, 42, 43], did not fall in the go‐zone and were rated below the overall mean for perceived equity. It is possible that repeating this study with youth might produce different rankings or a different set of strategies altogether. The absence of youth perspectives represents a gap, particularly given that youth engagement is essential to effective implementation of adolescent health interventions [44, 45]. Future research should seek youth perspectives and voices through age‐appropriate participatory methods to ensure that their experiences and ideas inform intervention design and implementation.

Third, to reduce response burden and the risk of identifying individuals in the rural communities where this study was conducted, we collected age and duration of residence at the sorting and rating stages but did not ask about parental status, community role, or gender. These characteristics may have influenced perceptions of the impact or equity of proposed strategies, but we cannot assess whether ratings differed by these characteristics or whether particular groups were over‐ or underrepresented among raters. We therefore cannot fully evaluate the representativeness of the study sample or differences in ratings by participant characteristics. The findings should not be interpreted as a representative community survey, but rather as an aggregate view of a modest number of engaged community members.

Fourth, ratings were compressed toward the upper end of both scales. This may reflect that the proposed strategies were broadly viewed as potentially beneficial and equitable, leaving relatively little differentiation among statements. Because go‐zone placement is based on whether a statement falls above the overall mean for both dimensions, small differences in ratings can affect whether individual statements fall inside or outside the go‐zone. Individual statements near the go‐zone boundary should therefore be interpreted flexibly and considered alongside other factors, such as community interest, acceptability, and feasibility, when identifying priorities for action.

Finally, this study was conducted online, requiring participants to have Internet access and digital literacy to navigate the GroupWisdom platform. While public spaces in rural areas provide Internet access and participants could use mobile devices, this requirement may have limited participation and potentially excluded perspectives from individuals with limited technology literacy or access.

## 6 Conclusions

This GCM study documented adult rural stakeholder perspectives on youth mental health intervention priorities. Participants rated strategies related to school‐based services, affordability, and mental health education highly for both perceived impact and perceived equity. The frequency with which participants identified workforce‐related needs, particularly providers trained in child and adolescent mental health, was notable. Findings from this study may inform future intervention planning in rural settings, though implementation feasibility and effectiveness of these strategies require further investigation.

## Funding

This project was supported by a pilot grant from the Institute for Research and Education to Advance Community Health (IREACH) at Washington State University. This project was also supported by the Health Resources and Services Administration (HRSA) of the US Department of Health and Human Services (HHS) under grant number UB6HP31690 titled Public Health Training Centers.

The information or content and conclusions presented in this publication are those of the authors and should not be construed as the official position or policy of, nor should any endorsements be inferred by IREACH, HRSA, HHS, or the US Government. The funders had no role in the study design, data collection, data analysis, writing, or decision to submit this research for publication.

## Conflicts of Interest

The authors declare no conflicts of interest.

## Supporting information




**Supporting File**: jrh70227‐sup‐0001‐SuppMat.docx
