# Supplementary material for: Adult Community Perspectives on Improving Rural Youth Access to Mental Health Services: A Participatory Group Concept Mapping Study
Source: J Rural Health. 2026 Oct 9;42(4):e70227. doi: 10.1111/jrh.70227 (PMC13650491; doi:10.1111/jrh.70227)
Supplement: Supplementary file 1 [file JRH-42-0-s001.docx]

**Supplemental** **Figure 1**. Point map illustrating the conceptual similarities of statements through spatial arrangement. Gray, numbered points on this map illustrate two-dimensional spatial relationship among the 79 statements. Closer points indicate more conceptually similar statements.

| **Supplemental Table 1.** Rating prompt provided to participants within GroupWisdom. | |
| --- | --- |
| Impact rating | **How much impact would this strategy have on increasing youth access to mental health services in your community?**  Please rate each statement on **the impact of the strategy in increasing youth access to mental health** in your rural northeastern Washington community. Rate each item on a scale from 1 to 5, with 1 being "no/little impact" and 5 being "very large impact."  For the purposes of this study, we want. you to think about your community as a whole, not only your personal experience(s). |
| Equity rating | **How equitable would this strategy be on increasing youth access to mental health services in your community?**  Please rate each statement on its **equity in increasing access to mental health services for all youth** in your rural northeastern Washington community. Rate each item on a scale from 1 to 5, with 1 being "not at all equitable" and 5 being "very equitable." Equity is defined as everyone having an equal opportunity to benefit from services.  For the purposes of this study, we want. you to think about your community as a whole, not only your personal experience(s). |

**Supplemental Table 2.** Pre-sorted, synthesized list of community-identified suggestions to help rural youth to receive care or support for emotional or mental health.

Assist youth in setting attainable life and emotional goals

Build community health centers where anyone can receive any form of health care in one place

Continue to educate and support students after graduation

Destigmatize mental health by creating spaces for kids and adults to talk about it

Develop and offer more youth-focused programs in communities

Develop hotlines for diverse mental health needs (not just suicide)

Develop programs for peer-peer support or peer listening

Disseminate positive examples and representation of/for those getting help

Educate the entire community about mental health (not just families with kids)

Employ more (or any) mental health counselors at schools

Employ more youth advocates

Encourage healthy activities within communities

Encourage more physical exercise in the community

Enhance multi-generational support in the communities

Ensure extra support for LGBTQ+ students

Formally train school staff in mental health screening

Help students more easily identify their need(s) for help

Improve access to basic services for families (housing, clothing, food)

Improve access to broadband internet for telehealth

Improve and expand anti-bullying programs

Improve communication and cooperation between schools and communities

Improve communication and relationships between the school and families

Improve community awareness of what help is available

Improve mental health resources for homeschool students

Improve school staff ability to recognize and help students with their mental health problems

Improve trust between patients and providers

Increase access to training on mental health signs and symptoms

Increase anonymity when seeking services

Increase availability of help

Increase awareness about available support in school

Increase awareness on domestic violence, pregnancy, and sexual assault in sexual education

Increase church and faith-based support networks

Increase community resources for mental health

Increase funding for services and to existing organizations

Increase mental health education for young children

Increase pay for counselors and mental health staff

Increase support from government agencies (CPS, DSHS)

Increase the healthcare workers in the community

Integrate mental health checks in annual child checkup/physical

Introduce a mentorship program for youth

Offer a public speaker forum to touch on mental health in schools and community

Offer age-appropriate support groups

Offer alternatives for juveniles involved in criminal justice system (join a sport, join a mentor program, community service etc.)

Offer external programs, trips, or experiences through schools

Offer extra attention and management of at-risk children

Offer local, 24/7 counseling services

Offer mandatory one-on-one counseling in school (e.g., twice a year)

Offer mental health assistance to youth via text

Offer mental health courses in school

Provide a crisis hotline in local Emergency Rooms

Provide access to services regardless of insurance type

Provide after-school programs for youth

Provide better/free transportation

Provide counseling services for teachers and school staff

Provide education about addiction and how to combat it

Provide education about how to foster and protect healthy relationships

Provide education on improving social-emotional skills

Provide families with management help and education

Provide free bussing to health clinics

Provide free health services, including mental health

Provide grief counseling through schools

Provide group therapy in schools

Provide life skill training

Provide mental health support to youth during school breaks

Provide more information about mental health through schools

Provide more information about mental health via social media platforms

Provide parents skills to help them be more supportive of and for their kids

Provide teen mental health training (e.g., Mental Health First Aid)

Reduce the costs of services (increase affordability)

Staff mental health clinics with providers who are trained to work with youth

Staff providers at locations where kids frequent

Take away the uncertainty (more/better scheduling)

Teach children coping skills

Teach stress management skills to youth

Teach youth what unhealthy relationships look like

Train law enforcement in mental health

Train school staff in trauma informed care or "Trauma Lens"

Treat and recognize mental health/illness the same as physical ailments

Develop and support school-based health clinics
